# Characteristics of Protein Profiling and Biomarkers in Aortic Regurgitation With Heart Failure

**DOI:** 10.1161/JAHA.125.047122

**Published:** 2026-04-07

**Authors:** Cheng‐Yao Ni, Hai‐Tao Hou, Hai‐Ge Zhao, Yi‐Ming Ni, Liang Ma, Guo‐Wei He

**Affiliations:** ^1^ Department of Cardiac Surgery, The First Affiliated Hospital Zhejiang University Hangzhou China; ^2^ Department of Cardiovascular Surgery, TEDA International Cardiovascular Hospital Tianjin University & Chinese Academy of Medical Sciences Tianjin China; ^3^ Tianjin Key Laboratory of Molecular Regulation of Cardiovascular Diseases and Translational Medicine Tianjin China

**Keywords:** ADIPOQ, aortic regurgitation, data independent acquisition, IGFBP7, proteomics, Proteomics, Valvular Heart Disease

## Abstract

**Background:**

Valvular heart disease, particularly aortic valve disease including stenosis and regurgitation, is a common heart disease. This study aimed to explore the protein profiling and the biomarkers in severe aortic valve disease and to provide new insights into the therapeutic strategy.

**Methods:**

Blood samples from 80 subjects were collected and analyzed by data independent acquisition technique in 3 comparisons (mild/moderate‐control, severe‐control, and severe–mild/moderate) and validated by ELISA. The diagnostic value of differentially expressed proteins associated with severe valvular heart disease was also evaluated by the receiver operating characteristic curve.

**Results:**

A total of 9976 peptides and 451 proteins were identified through liquid chromatography‐tandem mass spectrometry analysis. From these, 64 in mild/moderate‐control, 50 in severe‐control, and 50 in severe–mild/moderate comparisons were identified as differentially expressed proteins. IGFBP7 (insulin‐like growth factor‐binding protein 7; 5581.0±697.0 ng/mL), DSG1 (desmoglein‐1; 21.0±2.0 pg/mL), ADIPOQ (adiponectin; 26 686.0±3730 ng/mL), and JUP (junction plakoglobin; 10.2±0.6 ng/mL) levels in the severe group were significantly higher than that in the mild/moderate (*P*<0.05) group. Additionally, ADIPOQ and JUP levels in the severe group were also higher than that in control (*P*<0.001). Receiver operating characteristic curve analysis showed that IGFBP7, DSG1, JUP, and ADIPOQ had strong potential value to be associated with severe aortic valve disease.

**Conclusions:**

By constructing proteomics profile to identify the protein characteristics this study found that increased IGFBP7, DSG1, JUP, and ADIPOQ are the characteristics of proteins in patients with severe valvular heart disease. These findings provide new insight into the diagnosis and pathogenesis of valvular heart disease, particularly aortic valve disease.

**Nonstandard Abbreviations and Acronyms**
AR: aortic regurgitation
AVD: aortic valve disease
DEPs: differentially expressed proteins
GO: Gene Ontology
KEGG: Kyoto Encyclopedia of Genes and Genomes
LVDD: left ventricular end diastolic dimension
MS: mass spectrum
NYHA: New York Heart Association
VHD: valvular heart disease

**Clinical Perspective**

**What Is New?**

- This study for the first time reveals that the severity of valvular heart disease correlates to the plasma protein profiling, revealing the significance of the protein changes in aortic regurgitation.
- The increased IGFBP7 (insulin‐like growth factor‐binding protein 7), DSG1 (desmoglein‐1), JUP (junction plakoglobin), and ADIPOQ (adiponectin) have the role of predicting the severity of the cardiac dysfunction in the patients with severe valvular heart disease.

**What Are the Clinical Implications?**

- Our findings provide new insights into the diagnosis and pathological changes in valvular heart disease by plasma protein profiling and predict disease progression, particularly in aortic regurgitation.

Valvular heart disease (VHD) usually presents a process of chronic development and results in valvular damage.^1^ The most common valve diseases are calcific aortic stenosis and aortic regurgitation (AR).^2^ More than 40 million people are affected by either mitral or aortic valve disease (AVD) worldwide.^3^ AR can be caused by primary disease of the aortic valve cusps or abnormalities of the aortic root and ascending aortic geometry.^4^ Acute AR may lead to rapid cardiac decompensation and early death.^5^ Chronic severe AR can lead to progressive enlargement of the aortic root and further worsening of AR over time.^5^ Further, AVD is often associated with mitral valve disease, particularly in rheumatic heart valve disease. The risk factors for either operative mortality^6^ or long‐term survival^7^, ^8^ were reported.

Severe AR can produce massive left ventricular (LV) volume overload and progressive chamber dilation^5^ and severely enlarged LV may increase the operative risk. Therefore, surgery should be considered before the LV ejection fraction falls below 55% or the left ventricular end diastolic dimension (LVDD) reaches 55 mm.^5^

Studies on calcific AVD^9^ and aortic stenosis^10^, ^11^ have used proteomics to investigate their underlying mechanisms and pathophysiological alterations. We have identified the protein characteristics in various VHDs including rheumatic and degenerative valvular disease.^12^ Further, we have also identified the role of peroxisome proliferator‐activated receptor pathway in atrial fibrillation associated with VHD.^13^, ^14^

Nevertheless, the effect of the severity of the valvular disease on protein profiling has not been explored. Such investigation may facilitate the identification of protein biomarkers useful for monitoring disease progression.

The present study aimed to explore biomarkers associated with severe VHD by using proteomics methods and provide new insight of therapeutic strategy of severe VHD.

## METHODS

### Data Availability Statement

Data underlying this study will be shared on reasonable request to the corresponding author.

### Study Design and Population

The patients and the control subjects were divided into 3 groups:

Group I: healthy controls (n=10 for proteomics and n=26 for validation).

All in New York Heart Association (NYHA) class: I.

Group II: patients with mild/moderate VHD (LVDD ≤65 mm) (n=10 for proteomics and n=27 for validation).

NYHA class: I (0), II (19, 70.4%), III (4, 14.8%), IV (4, 14.8%).

Group III: patients with severe VHD (LVDD >65 mm) (n=10 for proteomics and n=27 for validation).

NYHA class: I (0), II (8, 29.6%), III (13, 48.1%), IV (6, 22.2%).

All the patients have AR. All patients in the severe group had severe AR, and the mild/moderate group comprised 8 patients with moderate AR and 19 with severe AR.

Table S1 shows the operation procedure, including aortic valve replacement for AR (54) and aortic stenosis (8), either alone or with concomitant mitral valve replacement. The cohort of 54 patients with aortic valve replacement included 2 patients with diabetes and hypertension and 19 patients with hypertension. Among these 54 patients, 8 had aortic stenosis. Of these, 6 were in the mild/moderate group and 2 in the severe group.

LVDD was determined by cardiac echocardiography. The mean transvalvular velocity in the mild/moderate group was 4.5±0.1 m/s (individual values: 4.1, 5.0, 4.5, 4.2, 4.8, 4.3). The 2 patients in severe group had the velocity of 5.2 and 3.1 m/s. The mean peak systolic pressure gradient in the mild/moderate group was 6.7±0.2 mm Hg (individual values: 6.8, 5.7, 7.0, 6.7, 6.8, 7.3). The 2 patients in the severe group had a gradient of 5.0 and 5.5 mm Hg. The mean aortic valve area in the mild/moderate group was 0.9±0.2 cm^2^ (individual values: 1.2, 0.6, 1.0, 0.4, 0.7, 1.4). The 2 patients in the severe group had an area of 0.4 and 1.6 cm^2^.

A total of 80 subjects were enrolled. Among them, each of the 30 subjects (10 per group) donated 2 samples; one was used for the discovery‐phase proteomics analysis (Stage I) and the other was used for validation phase. Therefore, the total number of samples was 110 from the 80 subjects. For the validation, a new larger cohort of individuals (n = 50) was added to enhance the accuracy of the results. Therefore, there were 80 subjects (control: n=26, mild/moderate: n=27, severe: n=27) in the validation phase for ELISA measurements (Stage II). The characteristics of the patients are presented in Table S2.

**Figure 1:**
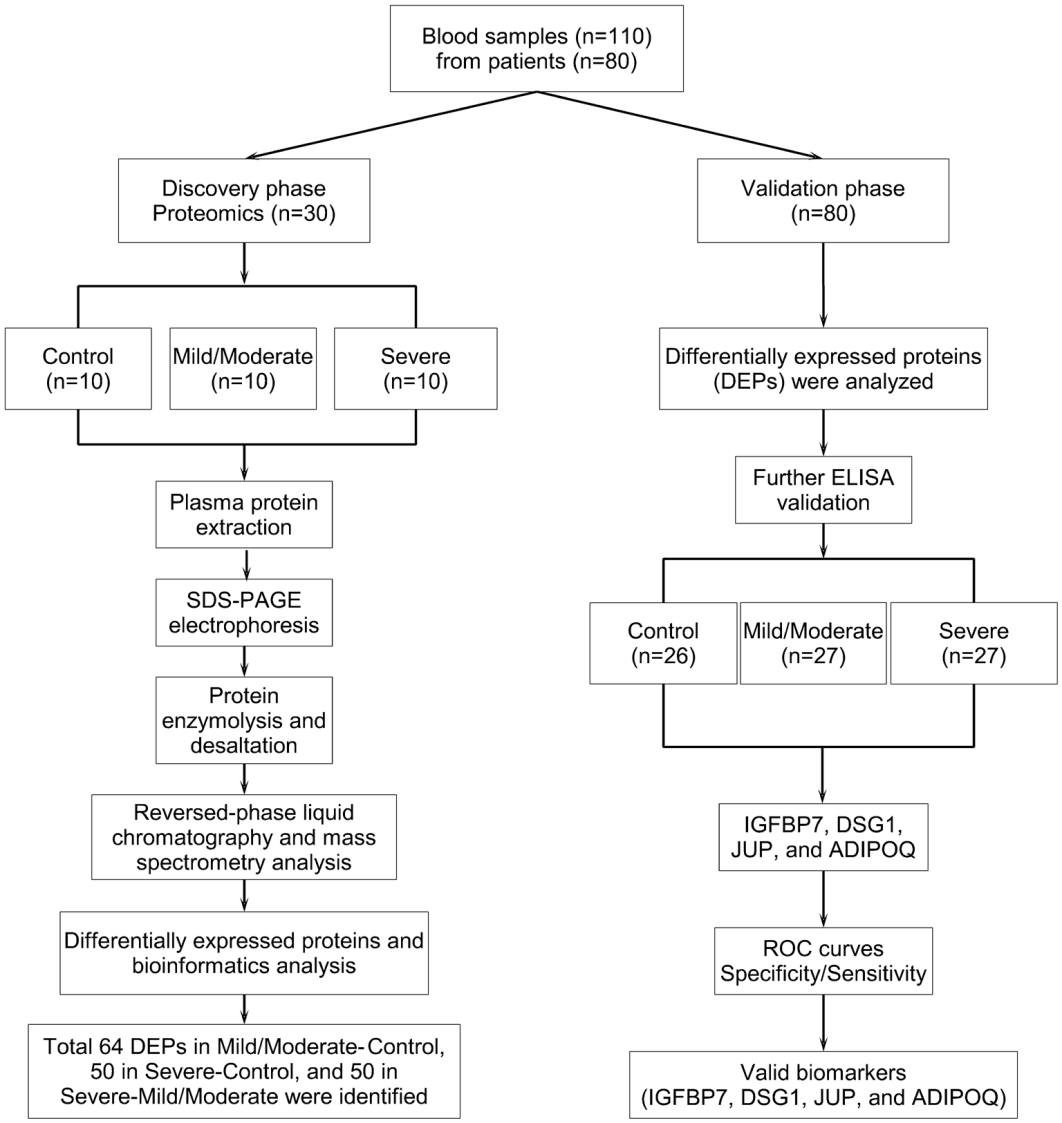
Consolidated Standards of Reporting Trials diagram of the study. ADIPOQ indicates adiponectin; DEP, differentially expressed protein; DSG1, desmoglein‐1; IGFBP7, insulin‐like growth factor‐binding protein 7; JUP, junction plakoglobin; and ROC, receiver operating characteristic.

The experimental design of the study is depicted in Figure 1. The protocol was approved by the Ethics Committee of the TEDA International Cardiovascular Hospital, Tianjin University and the First Affiliated Hospital, Zhejiang University School of Medicine. Informed consent was obtained. All samples were collected in accordance with the principles outlined in the Declaration of Helsinki.

### Sample Preparation

Blood samples were obtained by EDTA‐containing vacutainer tube and centrifuged at 2500 rpm (1000*g*) for 10 minutes. The upper plasma was separated and preserved in −80 °C until use.

### The First Stage: Proteomics Study

#### Data Independent Acquisition Spectral Library and Mass Spectrometric Acquisition

The peptide of pooled samples was fractionated by 1100 High Performance Liquid Chromatography System (Agilent, USA). Mobile phases A (2% acetonitrile in high performance liquid chromatography water) and B (90% acetonitrile in high performance liquid chromatography water) were used for reverse phase gradient. The solvent gradient was set as follows: 0 to 10 minutes, 98% A; 10 to 10.01 minutes, 98% to 95% A; 10.01 to 37 minutes, 95% to 80% A; 37 to 48 minutes, 80% to 60% A; 48 to 48.01 minutes, 60% to 10% A; 48.01 to 70 minutes, 10% A; 58 to 58.01 minutes, 10% to 98% A; 58.01 to 63 minutes, 98% A. Tryptic peptides were separated at an fluent flow rate of 250 μL/min and monitored at 210 nm. Samples were collected for 8 to 60 minutes, and eluent was collected in centrifugal tube every minute in turn. Samples were recycled in this order until the end of gradient process. The separated peptides were lyophilized for mass spectrometry.

Mass spectrometry analysis was performed by Q‐Exactive HF mass spectrometer (Thermo Fisher Scientific, USA) with a Nanospray Flex source (Thermo Fisher Scientific, USA). Samples were loaded and separated by a C18 column (50 cm×75 μm) on an EASY‐nLC 1200 system (Thermo Fisher Scientific, USA). The flow rate was 300 nL/min and the liner gradient was 90 minutes (0~60 minutes, 8%–25% B; 60~79 minutes, 25%–45% B; 79~80 minutes, 45%–100% B; 80~90 minutes, 100% B; mobile phase A=0.1% formic acid in water and B=0.1% F formic acid A in 80% acetonitrile).

Full mass spectrum (MS) scans were acquired in the mass range of 350 to 1650 m/z with mass resolutions of 120 000. The 20 most intense peaks in MS were fragmented with higher‐energy collisional dissociation with collision energy of 27. MS/MS spectra were obtained with a resolution of 30 000 and a maximum injection time of 80 ms. The Q Exactive HF dynamic exclusion was set for 40.0 seconds and run under positive mode.

#### Bioinformatics Analysis

Functional annotation and enrichment analyses were performed. Venn diagrams, principal component analysis, Gene Ontology (GO; biological process, cellular compartment, and molecular function) enrichment, and Kyoto Encyclopedia of Genes and Genomes (KEGG) were applied to analyze the functions of differentially expressed proteins (DEPs). Terms with *P*<0.05 were considered statistically significant. Hierarchical clustering heatmaps were generated using the pheatmap package of R (v3.6.1) language.

### The Second Stage: Validation Study

#### ELISA of Insulin‐Like Growth Factor‐Binding Protein 7, Desmoglein‐1, Adiponectin, and Junction Plakoglobin

To validate the proteomic findings, the expression levels of 4 key protein candidates (IGFBP7 [insulin‐like growth factor‐binding protein 7], DSG1 [desmoglein‐1], ADIPOQ [adiponectin], and JUP [junction plakoglobin]) were quantified by ELISA. The assays were performed using specific commercial kits (Cusabio Biotech, China) according to the manufacturer's instructions. Each sample was measured in duplicate to account for technical variability and ensure result reliability. The absorbance of each well was measured at 450 nm after incubation and reaction steps. The concentration of each sample was calculated based on the standard curve and its corresponding absorbance value.

### Statistical Analysis

Data were analyzed using SPSS 20.0 software (SPSS Inc., Chicago, IL, USA) and GraphPad Prism (version 7, GraphPad Software Inc., San Diego, CA). Data were represented as mean±SEM. *P* values were adjusted using the Benjamini–Hochberg false discovery rate method, and proteins with an adjusted *P*<0.05 and fold change >1.5 or <0.67 were considered as DEPs. The significance of GO terms and pathways was expressed with *P*<0.05. Hierarchical clustering was used to demonstrate the relationships using DEPs. One‐way ANOVA was used for comparisons among three groups, followed by Bonferroni correction for post hoc analysis.

## RESULTS

### The First Stage: Proteomics Study

#### Proteomics Profiling

**Figure 2:**
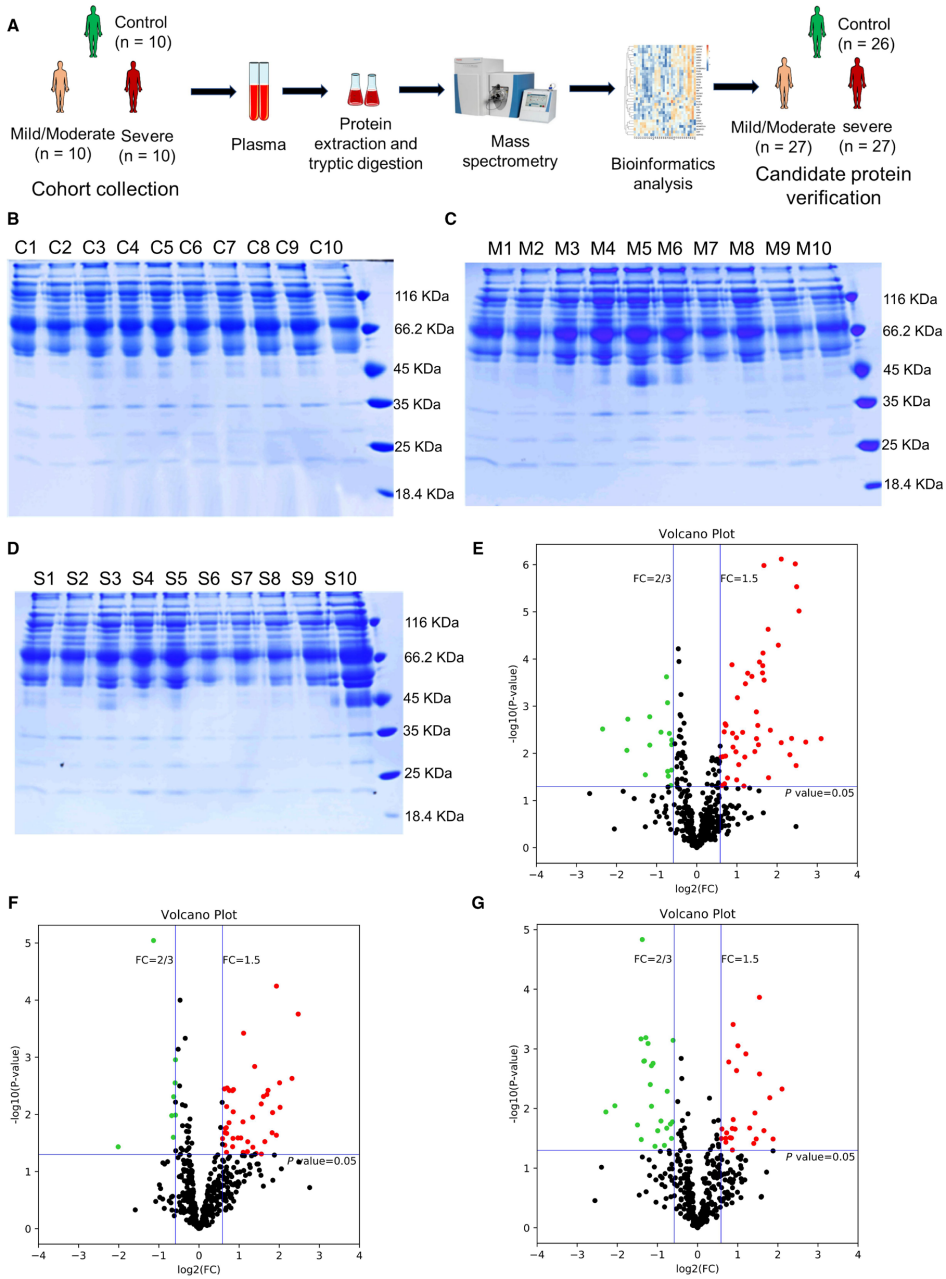
Overview of proteomics using data independent acquisition method. **A**, The schematic overview of the experimental approach. **B–D**, Evaluation of sample consistency by sodium dodecyl sulfate‐polyacrylamide gel electrophoresis technique. **E–G**, Volcano plot representing the profile of all the quantified proteins. Red, green, and black dots represent upregulation, downregulation, and no significant change, respectively.

**Figure 3:**
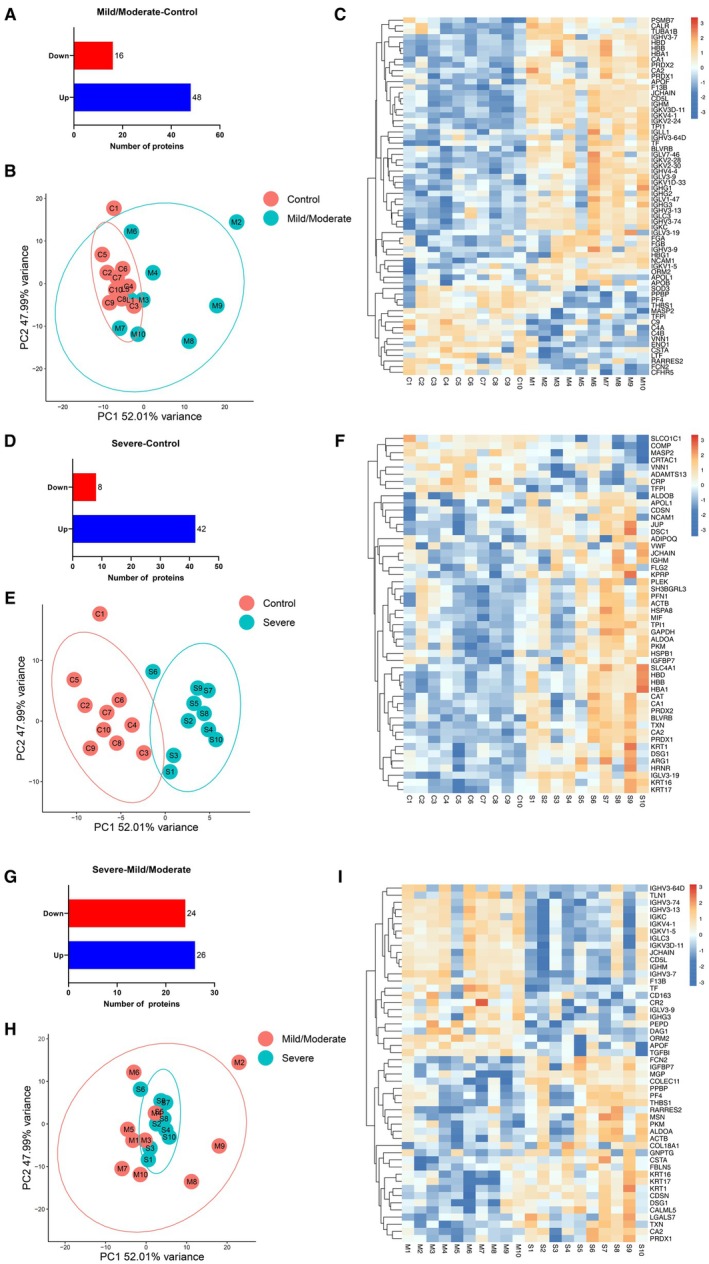
Analyses of differentially expressed proteins. **A**, **D**, and **G**, Principal component analysis reveals a relative distinction of all the samples. **B**, **E**, and **H**, Up‐regulated and down‐regulated proteins (*P*<0.05, fold change >1.5) in mild/moderate‐control, severe/control, and severe–mild/moderate. **C**, **F**, and **I**, Heatmap displaying the relative abundances in each comparison using all the differentially expressed proteins. PC indicates principal component.

Figure 1 is the flow chart of the experimental protocol. Figure 2A shows the overview of proteomics using the data independent acquisition method. Sodium dodecyl sulfate‐polyacrylamide gel electrophoresis technique shows that the samples in the 3 groups had consistent repeatability (Figure 2B through 2D). Figure 2E through 2G shows the profile of all the quantified proteins. A total of 9976 peptides and 451 proteins from the liquid chromatography‐tandem mass spectrometry analysis were identified. The principal component analysis (Figure 3A, 3D, and 3G) reveals a relative distinction among the samples of 3 groups.

#### Bioinformatics Analysis and Pathway Enrichment

Of the identified proteins, 14.2% (64 of 451, 48 upregulated and 16 downregulated, Figure 3B) in mild/moderate‐control, 11.1% (50 of 451, 42 upregulated and 8 downregulated, Figure 3E) in severe‐control, and 11.1% (50 of 451, 26 upregulated and 24 downregulated, Figure 3H) in severe–mild/moderate were screened as DEPs. Using DEPs, the heatmap (Figure 3C, 3F, and 3I) shows the relative abundance in each comparison group.

**Figure 4:**
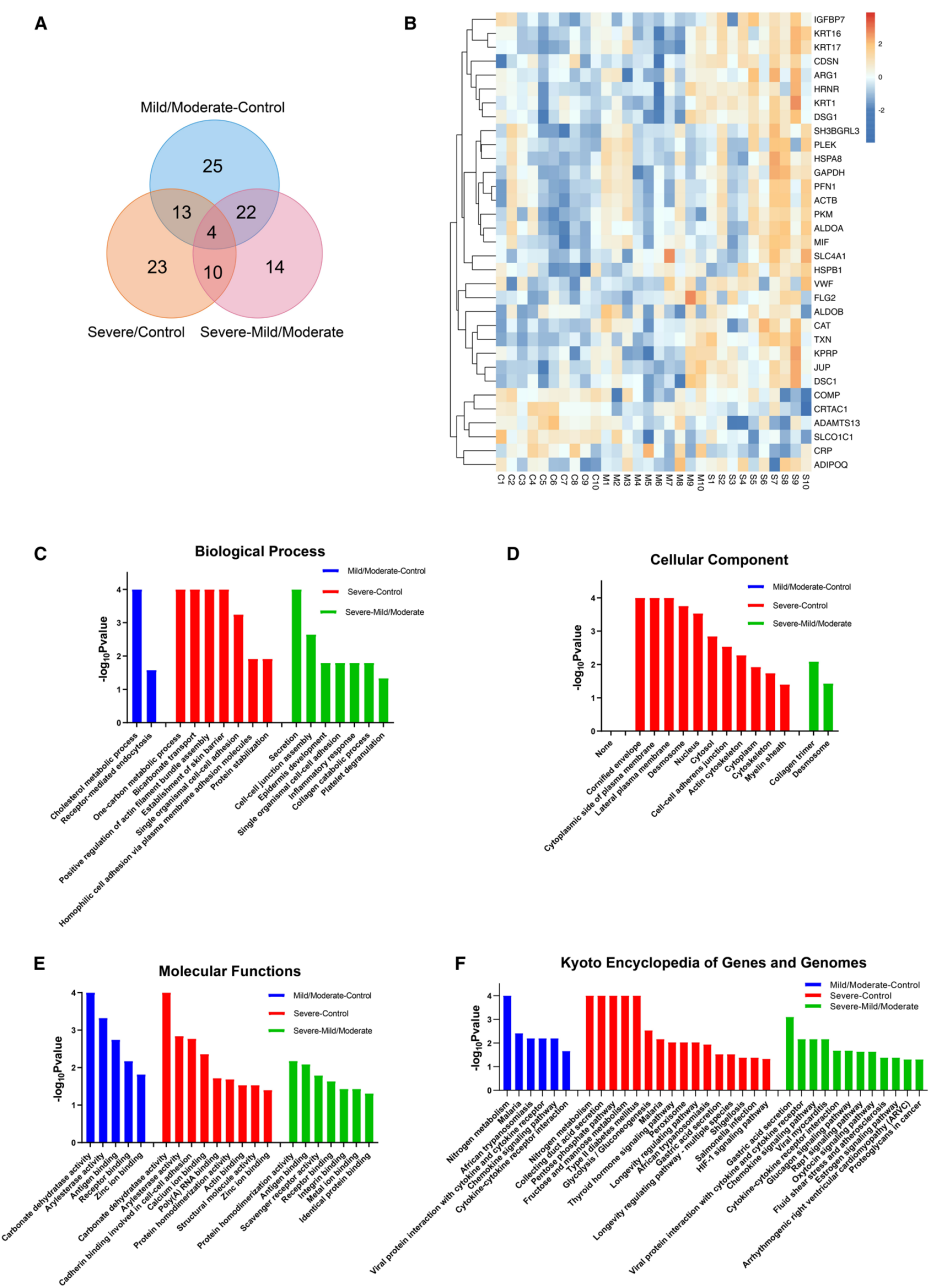
Bioinformatics analyses among mild/moderate‐control, severe/control, and severe–mild/moderate. **A**, Venn diagram showing the overlap of DEPs on the basis of 3 comparisons. A total of 10 proteins (ALDOA, KRT1, KRT16, TXN, PKM, ACTB, DSG1, KRT17, CDSN, and IGFBP7) show significant differences in severe‐control and severe–mild/moderate, but not in mild/moderate‐control. **B**, Heatmap showing the relative abundances of 14 DEPs. **C–F**, Significantly different Gene Ontology categories (biological process, cell component, and molecular function) and Kyoto Encyclopedia of Genes and Genomes pathways among 3 comparisons. ACTB indicates actin beta; ALDOA, aldolase A; CDSN, corneodesmosin; DEP, differentially expressed protein; DSG1, desmoglein‐1; IGFBP7, insulin‐like growth factor‐binding protein 7; KRT, keratin; PKM, pyruvate kinase; and TXN, thioredoxin.

Venn diagram shows (Figure 4A) the overlap of DEPs based on 3 comparisons. There are significant differences regarding 4 proteins (CA2 [carbonic anhydrase 2], JCHAIN [joining chain], IGHM [immunoglobulin heavy constant mu], and PRDX1 [peroxiredoxin‐1]) in all 3 comparisons. A total of 10 proteins (ALDOA [aldolase A], KRT1 [keratin 1], KRT16, TXN [thioredoxin], PKM [pyruvate kinase], ACTB [actin beta], DSG1, KRT17, CDSN [corneodesmosin], and IGFBP7) show significant differences in severe‐control and severe–mild/oderate but not in mild/moderate‐control. The heatmap of relative abundances of these 14 DEPs is shown in Figure 4B.

GO analyses including biological process, cellular component, and molecular functions were also performed using DEPs. Significant GO terms (*P*<0.05 and including at least 2 DEPs) were compared (Figure 4C through 4E).

Regarding biological process (Figure 4C), there are significant differences only in cholesterol metabolic process and receptor‐mediated endocytosis between mild/moderate and control. However, up to 7 terms (1‐carbon metabolic process, bicarbonate transport, positive regulation of actin filament bundle assembly, establishment of skin barrier, single organismal cell–cell adhesion, homophilic cell adhesion via plasma membrane adhesion molecules, and protein stabilization) were found in severe‐control. In particular, 7 terms (secretion, cell–cell junction assembly, epidermis development, single organismal cell–cell adhesion, inflammatory response, collagen catabolic process, and platelet degranulation) were found between severe and mild/moderate. Similarly, for cellular compartment (Figure 4D), up to 11 terms in severe‐control and 2 terms in severe‐mild/moderate were found. There were no differences (*P*<0.05 and including at least 2 DEPs) in mild/moderate‐control. Additionally, 9 terms in severe‐control, 5 terms in mild/moderate‐control, and 7 terms in severe–mild/moderate were found to have differences with regard to molecular function (Figure 4E). The specific GO enrichment information of various classifications of proteins was found in Figure S1.

Furthermore, the comparisons on KEGG (Figure 4F) were also performed. Every significant pathway involved at least 2 proteins. Especially, 8 pathways, including viral myocarditis, glucagon signaling pathway, rap1 signaling pathway, oxytocin signaling pathway, fluid shear stress and atherosclerosis, estrogen signaling pathway, arrhythmogenic right ventricular cardiomyopathy, and proteoglycans in cancer were found only between severe and mild/moderate. The specific KEGG enrichment information of all various classifications of proteins was found in Figure S2.

### The Second Stage: Validation Study

#### Validation of the Candidate Proteins by ELISA

**Figure 5:**
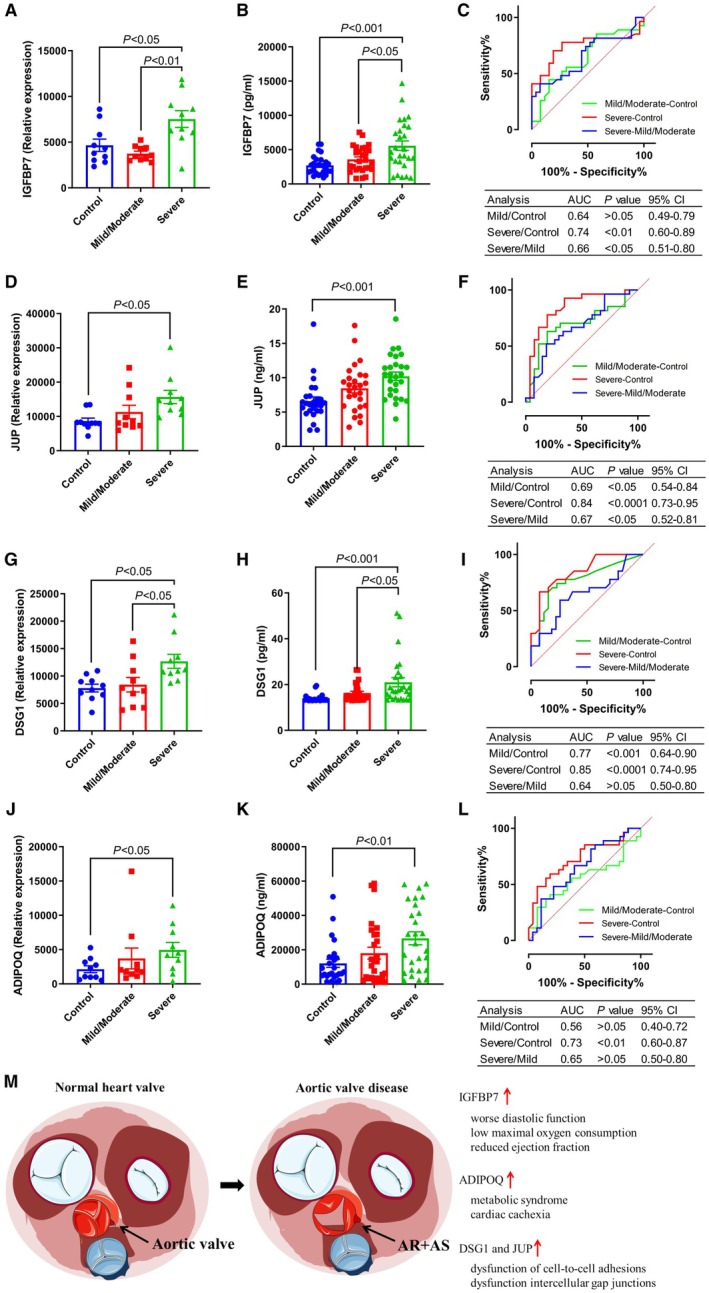
Validation and discrimination analyses of 4 differentially expressed protein candidates. **A**, **D**, **G**, and **J**, Discovery of IGFBP7, DSG1, JUP, and ADIPOQ by data independent acquisition technology. **B**, **E**, **H**, and **K**, Validation by enzyme linked immunosorbent assay in a new cohort of patients. **C**, **F**, **I**, and **L**, Receiver operating characteristic analyses show that these 4 proteins have strong and potential value of predicting severe aortic valve disease. **M**, Schematic diagram to illustrate the roles of IGFBP7, ADIPOQ, DSG1, and JUP to aortic valve. Elevated IGFBP7 is correlated to worse diastolic function, low maximal oxygen consumption, and reduced ejection fraction. Higher ADIPOQ is related to metabolic syndrome and cardiac cachexia. Increased DSG1 and JUP are correlated to dysfunction of cell‐to‐cell adhesions and intercellular gap junctions. ADIPOQ indicates adiponectin; AR, aortic regurgitation; AS, aortic stenosis; AUC, area under the curve; DSG1, desmoglein‐1; IGFBP7, insulin‐like growth factor‐binding protein 7; and JUP, junction plakoglobin.

Further validation of IGFBP7, DSG1, JUP, and ADIPOQ were performed in the new cohort of patients by ELISA. Figure 5A, 5D, 5G, and 5J show the intensity of JUP, IGFBP7, ADIPOQ, and DSG1, selected from the omics phase by data independent acquisition technology.

Further validations of these 4 proteins were performed in a new cohort of patients by ELISA. The results showed that IGFBP7 level in severe (5581.0±697.0 ng/mL) was significantly higher than that in mild/moderate (3597.0±358.9 pg/mL, *P*<0.05) or control (2706.0±274.0 pg/mL, *P*<0.001) (Figure 5B). However, there were no significant differences between mild/moderate and control. Similarly, DSG1 in severe patients (21.0±2.0 pg/mL) was also significantly higher than that in mild/moderate (16.3±0.7 pg/mL, *P*<0.05) or control (13.9±0.3 pg/mL, *P*<0.001) (Figure 5H).

JUP level has only marked significance in severe patients (10.2±0.6 ng/mL) compared with control (6.6±0.6 ng/mL, *P*<0.001) (Figure 5E). In addition, the difference of ADIPOQ was also found only between severe (26 686.0±3730 ng/mL, *P*<0.01) and control (12 093.0±2354 ng/mL, *P*<0.01) (Figure 5K). However, the levels of these 2 proteins in mild/moderate (JUP: 8.5±0.7 ng/mL; ADIPOQ: 18 012.0±3436.0 ng/mL) had no significant differences compared with severe or control.

### Diagnostic Performance of 4 Proteins

In general, receiver operating characteristic analysis shows that IGFBP7 and ADIPOQ have potential value of predicting severe AR. Regarding IGFBP7 (Figure 5C), the area under curve (AUCs) (95% CI) of mild/moderate‐control (*P*>0.05), severe‐control (*P*<0.01), and severe‐mild/moderate (*P*<0.05) are 0.64 (0.49–0.79), 0.74 (0.60–0.89), and 0.66 (0.51–0.80), respectively. Similarly, the areas under curve of ADIPOQ (Figure 5L) in mild/moderate‐control (*P*>0.05), severe‐control (*P*<0.01), and severe–mild/moderate (*P*>0.05) are 0.56 (95% CI, 0.40–0.72), 0.73 (95% CI, 0.60–0.87), and 0.65 (95% CI, 0.50–0.80), respectively.

In addition, the areas under curve (95% CI) of JUP (Figure 5F) in mild/moderate‐control (*P*<0.05), severe‐control (*P*<0.0001), and severe–mild/moderate (*P*<0.05) are 0.69 (95% CI, 0.54–0.84), 0.84 95% CI, (0.73–0.95), and 0.67 (95% CI, 0.52–0.81), respectively. The areas under curve (95% CI) of DSG1 (Figure 5I) in mild/moderate‐control (*P*<0.001), severe‐control (*P*<0.0001), and severe–mild/moderate (*P*>0.05) are 0.77 (95% CI, 0.64–0.90), 0.85 (95% CI, 0.74–0.95), and 0.64 (95% CI, 0.50–0.80), respectively.

## DISCUSSION

The present study presents a protein profiling using data independent acquisition proteomics approaches and reveals the differences among severe AVD, mild/moderate AVD, and healthy control, which is related to the biological basis for the mechanism of AVD. Importantly, this study suggests that (1) severe AR had 50 DEPs in comparison with the mild/eoderate AR, reaving the severity of the AR correlates to the protein changes; (2) among the 50 DEPs, IGFBP7 and ADIPOQ significantly increased in the patients with severe AR and therefore these 2 proteins may be developed as biomarkers for the severity of AVD. Figure 5M summarizes the correlation between elevated IGFBP7, ADIPOQ, DSG1, and JUP and normal aortic valve or AVD.

It must be realized that the size of LVDD does not necessarily correlate with heart function. Cardiac hypertrophy could be physiological or pathological.^15^ In the early stage, hypertrophy could be a physiological compensation to a pathogen such as hypertension or AR, as seen in this study. The response of a particular individual (or the heart) may be different compared with others. This could be correlated to body size and the different tolerance of a particular individual. As seen in this study, Group II (the mild/moderate group) includes 14.8% NYHA class IV patients, whereas Group III (the severe group) contains 29.6% NYHA class II patients. However, on the other hand, there is a correlation between the LVDD and the severity of the pathological status of the heart. Also seen in our data, the proportion of patients in the more symptomatic classes (NYHA III and IV) increased from Group II (mild/moderate group, 29.6%) to Group III (severe group, 70.3%), a trend that aligns with the pathophysiological expectation that advanced ventricular remodeling is often associated with worsening functional status. Nevertheless, LVDD is a clinically convenient index because it is easily to find from a simple echocardiogram.

GO and KEGG analyses are effective methods for exploring the potential functions of DEPs. Previous studies have reported that the extracellular matrix plays a crucial role in aortic valve disease and regulates cells through various mechanisms, including the modulation of adhesion receptors and cytokines.^16^ In GO analysis (biological processes, cellular components, and molecular functions), we observed enrichment related to cell–cell adhesion and cell–cell junction (including JUP and DSG1) in both severe‐control and severe–mild/moderate comparisons. Dysfunction of cell–cell adhesions and intercellular gap junctions has been reported to contribute to cardiac abnormalities.^17^ Additionally, KEGG enrichment analysis highlighted cytokine–cytokine receptor interactions. These bioinformatics findings suggest that dysregulated cell–extracellular matrix interactions, particularly involving adhesion and intercellular gap junctions, may lead to the pathogenesis of aortic valve disease.

### IGFBP7

IGFBPs were initially considered to be the regulatory proteins of IGFs during environmental changes in blood circulation. However, in recent years, IGFBPs has been found to have many functions independent of IGFs in regulating transcription, inducing cell migration, and apoptosis.^18^

It has been reported^19^, ^20^ that single‐cardiomyocyte transcriptome and plasma proteome integrative analyses reveal that IGFBP7, which is a cytokine downstream of TGF‐β (transforming growth factor‐beta) and secreted from failing cardiomyocytes, plays roles in regulating cardiomyocyte homeostasis and cardiac fibrosis.

Further, IGFBP7 has also been proposed as a potential prognostic biomarker in heart failure.^19^, ^21^ Higher IGFBP7 in patients with heart failure with reduced ejection fraction was associated with worse clinical profile and an increased risk of adverse clinical outcomes.^21^ In fact, IGFBP7 circulating concentrations are significantly associated with myocardial diastolic abnormalities.^20^ Higher baseline IGFBP7 is modestly correlated with worse diastolic function and lower baseline maximal oxygen consumption.^20^ IGFBP7 has been proposed as a biomarker to provide an excellent diagnostic accuracy in the prediction of acute kidney injury in cardiac surgery^22^, ^23^ and is superior to that of serum creatinine.^22^

In this study, IGFBP7 in severe AR significantly increased in comparison to the control or mild AR group. Taken together, it is obvious that severe AR is often associated with heart failure and therefore increased IGFBP7 is a potential biomarker for severe AR associated with heart failure.

### ADIPOQ

ADIPOQ is an adipokine secreted by adipose tissue and is a 244‐amino acid peptide involved in fatty acid metabolism, as well as glucose regulation.^20^ ADIPOQ has numerous beneficial effects against cardiovascular diseases.^24^ Elevated values of ADIPOQ have been reported in patients with cardiac cachexia and may be prognostic in heart failure.^20^, ^24^ It was also reported that ADIPOQ is elevated due to ADIPOQ resistance in subjects with metabolic syndrome or heart failure.^25^ On the other hand, it was reported that ADIPOQ expression was significantly lower in metabolic syndrome group undergoing coronary artery bypass grafting compared with nonmetabolic syndrome patients without coronary artery disease undergoing heart valve surgery.^26^

Interestingly, in the present study we found significant higher concentration of ADIPOQ in severe AR compared with control, suggesting the importance of this protein in the pathological process of severe AR.

### DSG1 and JUP

Desmosomes are cell–cell adhesion structures and are essential for the integrity of various tissues.^27^ Genetic variants of desmosomal proteins involving 2 structural desmosomal proteins DSG2 and junction plakoglobin^27^, ^28^, ^29^ are known to cause dysfunction of cell‐to‐cell adhesions and intercellular gap junctions^17^ and may result in skin, hair, or cardiac abnormalities.^28^ Arrhythmogenic cardiomyopathy is a genetic disease causing arrhythmia, heart failure, and sudden cardiac death. Mutations of DSG2 and JUP are the major cause of arrhythmogenic cardiomyopathy.^30^, ^31^ Interestingly, to our knowledge, the role of DSG1 and JUP in severe AR with heart failure has not been reported.

Our hypothesis is based on the established association of DSG1 and autoimmune disorders.^32^ The association is known to present with heart failure and arrhythmias that have clinical manifestations closely related to severe valvular heart disease. This indirect pathological link led us to speculate that DSG1 may also play a role in the progression of severe heart failure. Furthermore, our bioinformatics analysis showed that DSG1 and JUP are always enriched in several relevant pathways at the same time, for instance, in “single‐organism cell‐cell adhesion” (GO:0016337), which supports a potential functional synergy between these proteins in disease. We acknowledge that novel associations often arise from areas with sparse prior literature. Although findings supported by existing reports provide valuable confirmation, biomarkers or mechanisms that are underexplored may hold significant, previously unrecognized importance.

### Limitations

The present study is a proteomics study that usually involves a limited number of patients due to the complexity of the proteomics analyses and the cost. However, the findings from this study may open a new direction for the diagnostic and prognostic strategy of severe AR with heart failure. In particular, the identification of the potential biomarkers from this study may help choosing optimal operating time and predicting the severity of the cardiac dysfunction in the patients with severe AR.

## CONCLUSIONS

By constructing proteomics profile to identify the protein characteristics this study found that increased IGFBP7, DSG1, JUP, and ADIPOQ are the characteristic proteins in the severe AVD. The increased IGFBP7, DSG1, JUP, and ADIPOQ are potential biomarkers for predicting the severity of the cardiac dysfunction in patients with severe AR. These findings provide new insight into the pathogenesis of the development of severe AVD. However, further investigation is necessary to validate their diagnostic value.

## Sources of Funding

This work was supported by grants from the National Natural Science Foundation of China [82370350 and 82170353]; Science Technology Department of Zhejiang Province [2023C03087], Tianjin Key Medical Discipline Construction Project [TJYXZDXK‐3‐036C] and Special Fund for High Quality Development Project.

## Disclosures

None.

## Supporting information

Tables S1–S2
Figures S1–S2
