# Supplementary material for: Characteristics of Protein Profiling and Biomarkers in Aortic Regurgitation With Heart Failure
Source: J Am Heart Assoc. 2026 Apr 7;15(8):e047122. doi: 10.1161/JAHA.125.047122 (PMC13279130; doi:10.1161/JAHA.125.047122)
Supplement: Supplementary file 1 — Tables S1–S2 Figures S1–S2 [file JAH3-15-e047122-s001.pdf]

SUPPLEMENTAL MATERIAL

Table S1. Operation procedures.

Operation procedure	Patients in Stage I: Proteomics				Patients in Stage II: Verification			
	Control (n=10)	Mild/Moderate (n=10)	Severe (n=10)	*P* value	Control (n=26)	Mild/Moderate (n=27)	Severe (n=27)	*P* value
AVR	0	8	9	>0.05	0	19	18	>0.05
AVR+MVR	0	2	1		0	8	9	

AVR, Aortic valve replacement; MVR, Mitral valve replacement

Table S2. Patient characteristics of the proteomics/validation cohorts among three groups (n=80).

Variables	Proteomics patients				Verification patients			
	Control (n=10)	Mild/Moderate (n=10)	Severe (n=10)	*P* value	Control (n=26)	Mild/Moderate (n=27)	Severe (n=27)	*P* value
Sex (male/female)	8/2	8/2	8/2	-	18/8	18/9	18/9	-
Age, y	58.1±1.9	55±3.0	60.9±1.9	>0.05	58.3±2.1	55.4±2.5	58.4±1.8	>0.05
AO (mm)	32.5±0.7	31.4±1.2	36.9±1.8	<0.05	29.6±0.7	31.3±0.8	34.8±1.2	<0.001
LVDd (mm)	46.8±1.4	56.8±1.3	73.9±2.2	<0.001	46.8±0.6	56.6±0.9	73.9±1.3	<0.001
LAd (mm)	37.8±1.2	39.9±2.3	36.8±2.2	>0.05	44.8±2.4	43.1±2.3	42.7±1.8	>0.05
LVEF (%)	64.9±1.9	55±3.0	60.9±1.9	<0.001	63.2±0.7	63.1±2.0	58.4±1.8	<0.001

AO, Aorta dimension; LVDd, Left ventricular end diastolic dimension; LAd, left atrial dimension; LVEF, left ventricular ejection fraction.

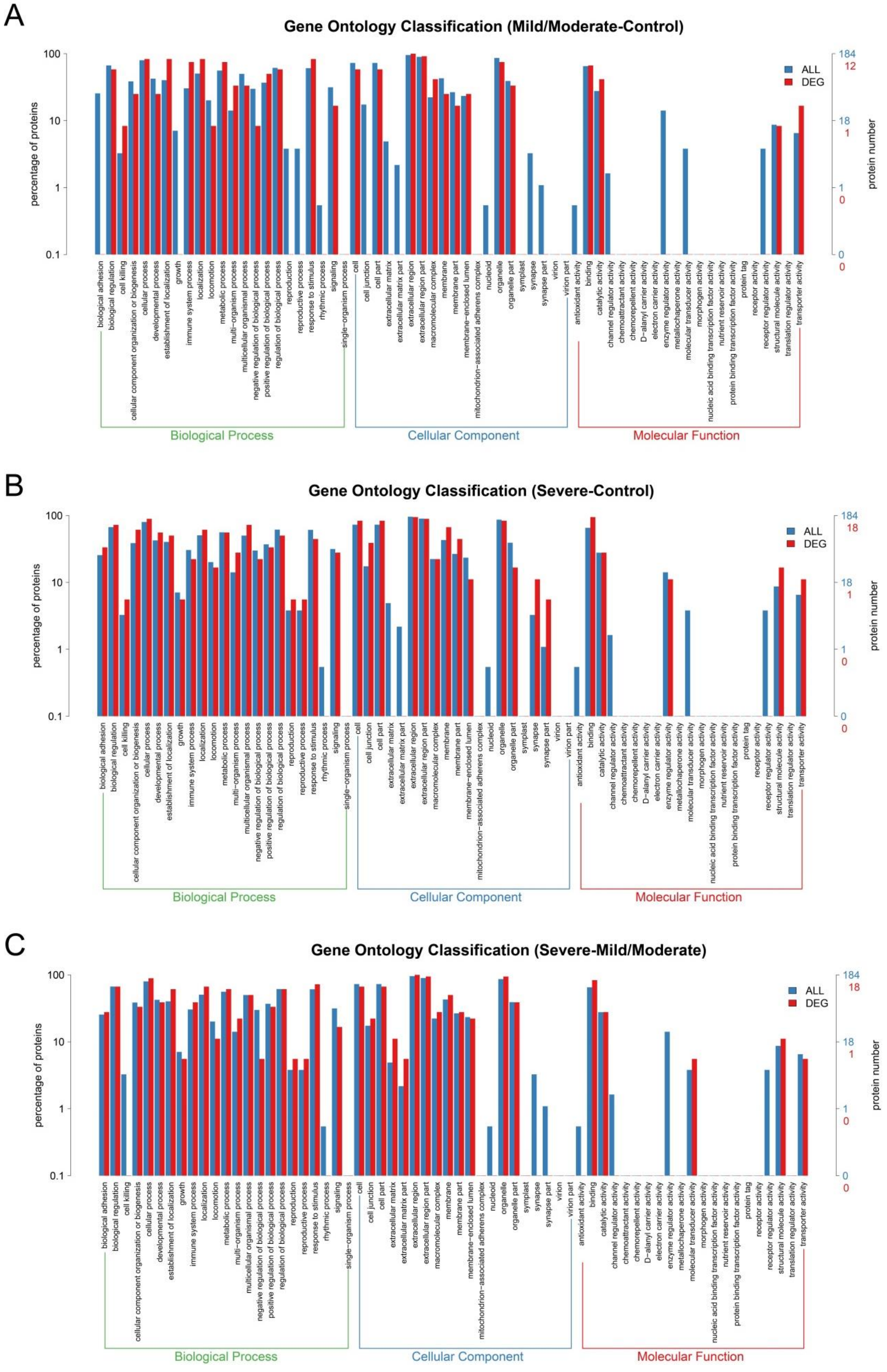
A
Gene Ontology Classification (Mild/Moderate-Control)
percentage of proteins
protein number
ALL
DEG
100
10
1
0.1
184
12
18
1
0
biological adhesion
biological regulation
cell killing
cellular component organization or biogenesis
cellular process
developmental process
establishment of localization
growth
immune system process
localization
locomotion
metabolic process
multi-organism process
multicellular organismal process
negative regulation of biological process
positive regulation of biological process
regulation of biological process
reproduction
reproductive process
response to stimulus
rhythmic process
signaling
single-organism process
cell
cell junction
cell part
extracellular matrix
extracellular matrix part
extracellular region
extracellular region part
macromolecular complex
membrane
membrane part
membrane-enclosed lumen
mitochondrion-associated adherens complex
nucleoid
organelle
organelle part
symplast
synapse
synapse part
virion
virion part
antioxidant activity
binding
catalytic activity
channel regulator activity
chemoattractant activity
chemorepellent activity
D-alanyl carrier activity
electron carrier activity
enzyme regulator activity
metallochaperone activity
molecular transducer activity
morphogen activity
nucleic acid binding transcription factor activity
nutrient reservoir activity
protein binding transcription factor activity
protein tag
receptor activity
receptor regulator activity
structural molecule activity
translation regulator activity
transporter activity
Biological Process
Cellular Component
Molecular Function
B
Gene Ontology Classification (Severe-Control)
18
C
Gene Ontology Classification (Severe-Mild/Moderate)

Figure S1-continued

D

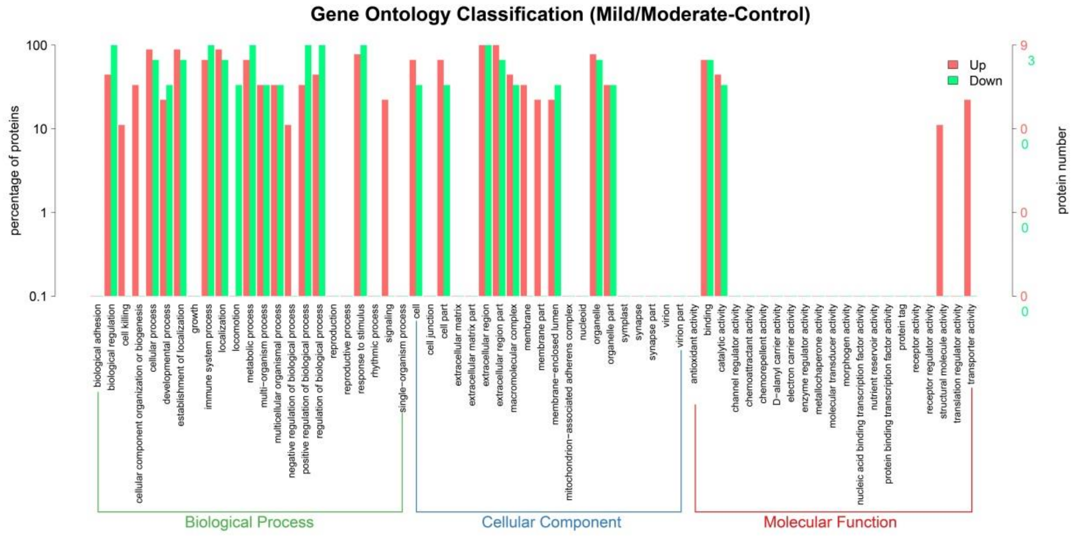

E

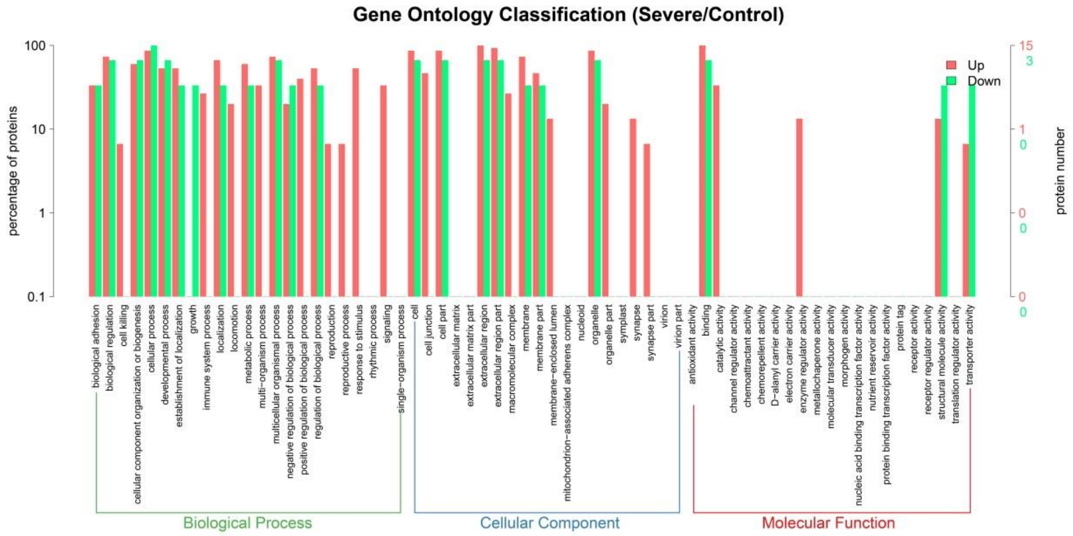

F

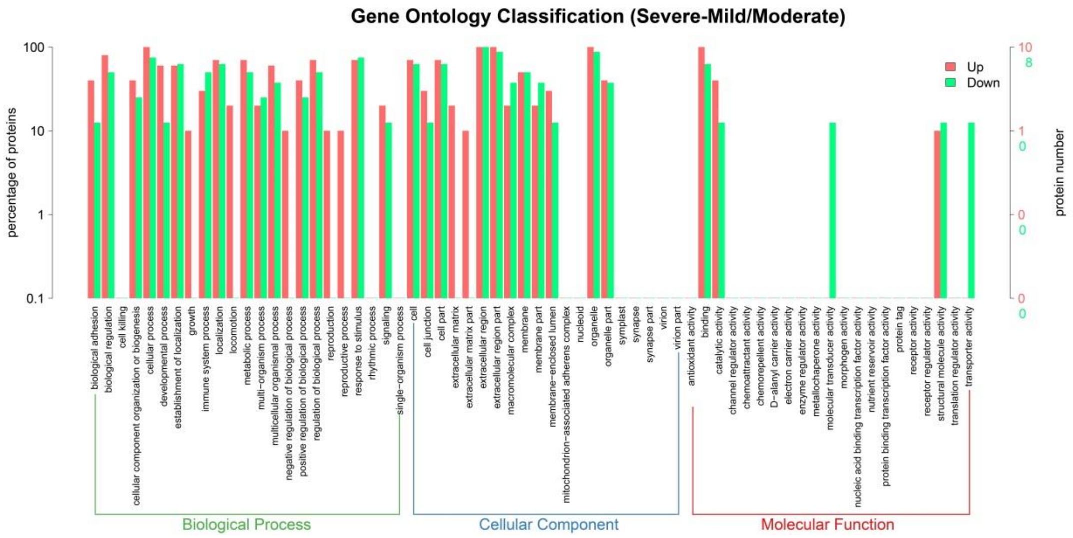

Figure S1. **Gene oncology (GO) specific enrichment analyses.**

A-C, using all identified proteins and differentially expressed proteins in Mild/Moderate-Control, Severe/Control, and Severe-Mild/Moderate, respectively.

D-F, using up-regulated and down-regulated proteins in Mild/Moderate-Control, Severe/Control, and Severe-Mild/Moderate, respectively.

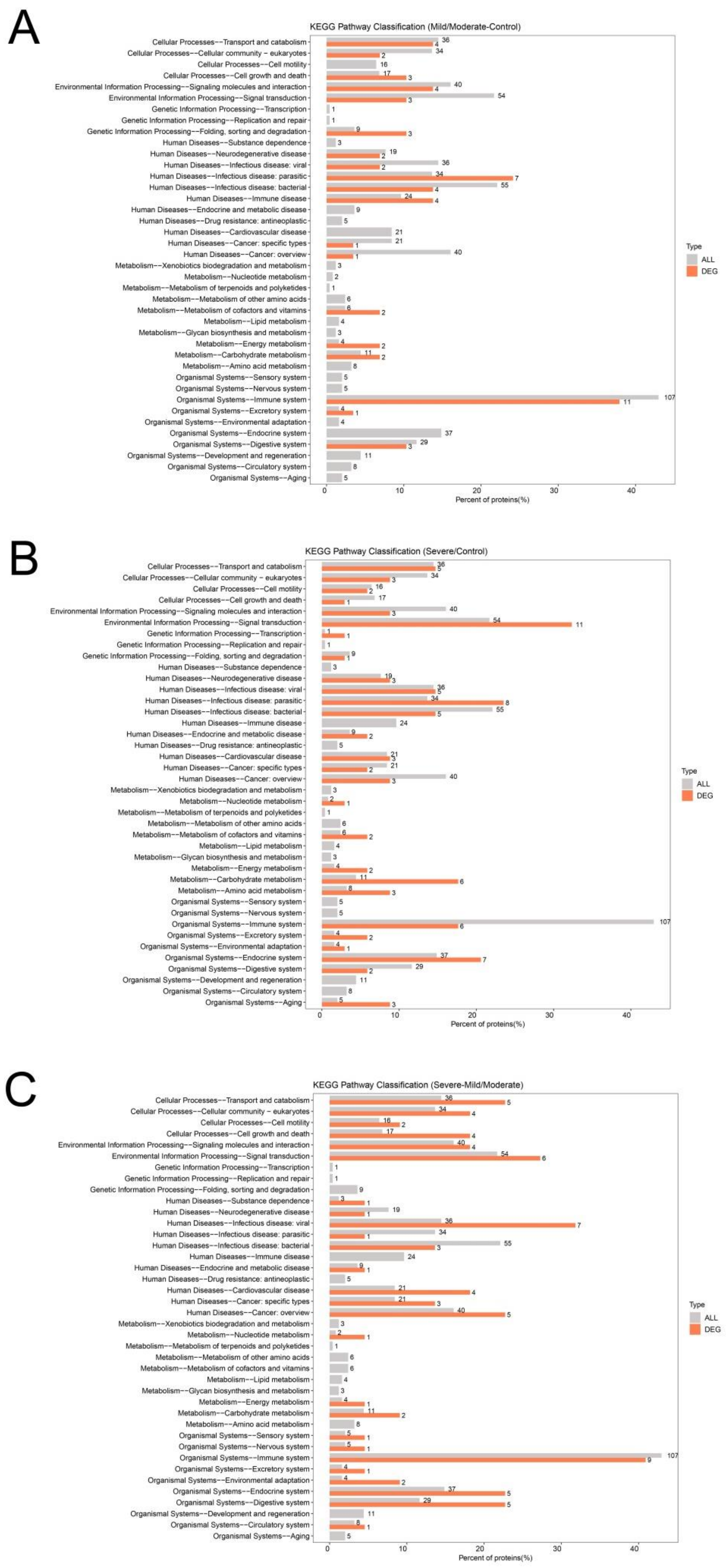
A
KEGG Pathway Classification (Mild/Moderate-Control)
Cellular Processes--Transport and catabolism
Cellular Processes--Cellular community - eukaryotes
Cellular Processes--Cell motility
Cellular Processes--Cell growth and death
Environmental Information Processing--Signaling molecules and interaction
Environmental Information Processing--Signal transduction
Genetic Information Processing--Transcription
Genetic Information Processing--Replication and repair
Genetic Information Processing--Folding, sorting and degradation
Human Diseases--Substance dependence
Human Diseases--Neurodegenerative disease
Human Diseases--Infectious disease: viral
Human Diseases--Infectious disease: parasitic
Human Diseases--Infectious disease: bacterial
Human Diseases--Immune disease
Human Diseases--Endocrine and metabolic disease
Human Diseases--Drug resistance: antineoplastic
Human Diseases--Cardiovascular disease
Human Diseases--Cancer: specific types
Human Diseases--Cancer: overview
Metabolism--Xenobiotics biodegradation and metabolism
Metabolism--Nucleotide metabolism
Metabolism--Metabolism of terpenoids and polyketides
Metabolism--Metabolism of other amino acids
Metabolism--Metabolism of cofactors and vitamins
Metabolism--Lipid metabolism
Metabolism--Glycan biosynthesis and metabolism
Metabolism--Energy metabolism
Metabolism--Carbohydrate metabolism
Metabolism--Amino acid metabolism
Organismal Systems--Sensory system
Organismal Systems--Nervous system
Organismal Systems--Immune system
Organismal Systems--Excretory system
Organismal Systems--Environmental adaptation
Organismal Systems--Endocrine system
Organismal Systems--Digestive system
Organismal Systems--Development and regeneration
Organismal Systems--Circulatory system
Organismal Systems--Aging
Percent of proteins(%)
Type
ALL
DEG
B
KEGG Pathway Classification (Severe/Control)
Percent of proteins(%)
Type
ALL
DEG
C
KEGG Pathway Classification (Severe-Mild/Moderate)
Percent of proteins(%)
Type
ALL
DEG

Figure S2-continued

D

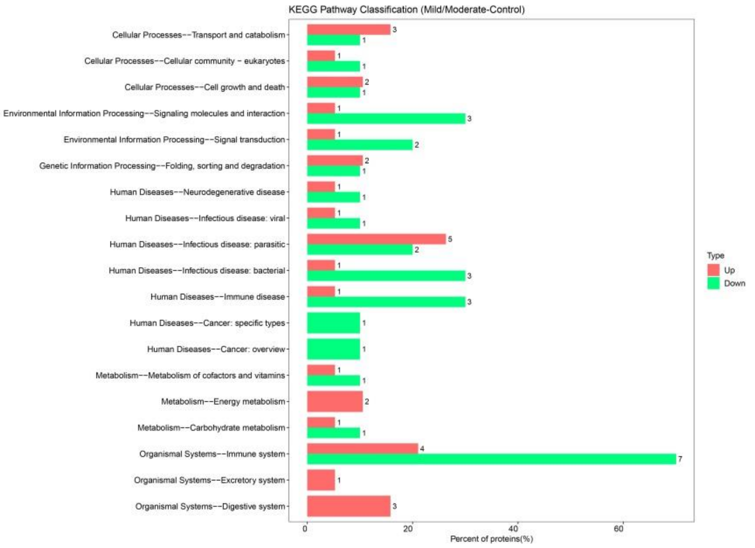

E

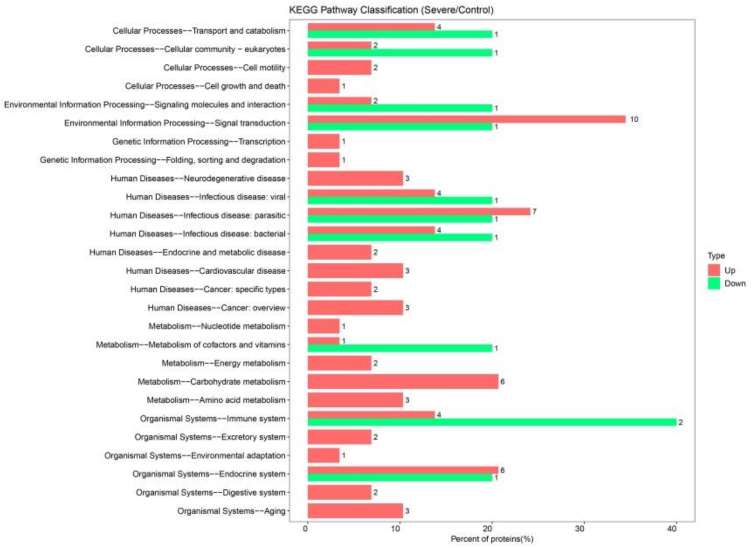

F

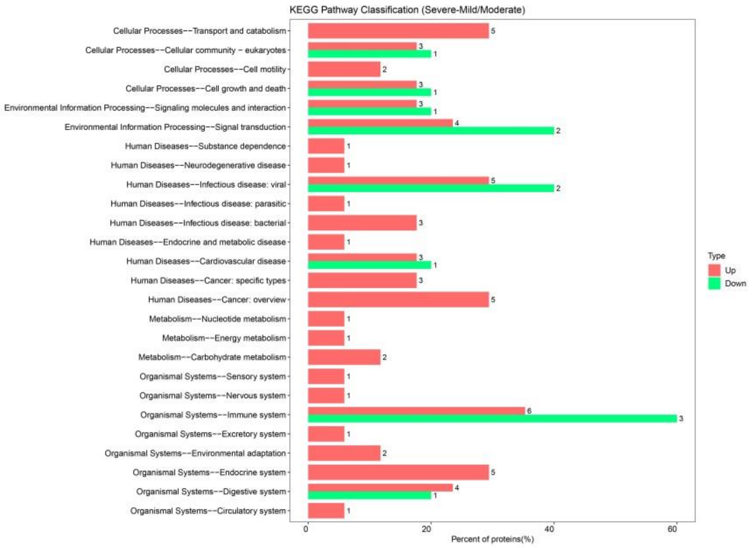

Figure S2. **Kyoto Encyclopedia of Genes and Genomes (KEGG) specific enrichment analyses.**

A-C, using all identified proteins and differentially expressed proteins in Mild/Moderate-Control, Severe/Control, and Severe-Mild/Moderate, respectively.

D-F, using up-regulated and down-regulated proteins in Mild/Moderate-Control, Severe/Control, and Severe-Mild/Moderate, respectively.
